# Microbiome Analysis from Paired Mucosal and Fecal Samples of a Colorectal Cancer Biobank

**DOI:** 10.3390/cancers12123702

**Published:** 2020-12-09

**Authors:** Ulrich Wirth, Debora Garzetti, Lara M. Jochum, Stefanie Spriewald, Florian Kühn, Matthias Ilmer, Serene M. L. Lee, Hanno Niess, Alexandr V. Bazhin, Joachim Andrassy, Jens Werner, Barbara Stecher, Tobias S. Schiergens

**Affiliations:** 1 Department of General, Visceral and Transplant Surgery, Ludwig-Maximilians-University Munich, Marchioninistr. 15, D-81377 Munich, Germany; Ulrich.Wirth@med.uni-muenchen.de (U.W.); Florian.Kuehn@med.uni-muenchen.de (F.K.); Matthias.Ilmer@med.uni-muenchen.de (M.I.); Serene.Lee@med.uni-muenchen.de (S.M.L.L.); Hanno.Niess@med.uni-muenchen.de (H.N.); Alexandr.Bazhin@med.uni-muenchen.de (A.V.B.); Joachim.Andrassy@med.uni-muenchen.de (J.A.); Jens.Werner@med.uni-muenchen.de (J.W.); 2 Max-Von-Pettenkofer Institute, Ludwig-Maximilians-University Munich, Pettenkoferstr. 9A, D-80336 Munich, Germany; Debora.garzetti@gmail.com (D.G.); Laramjochum@gmail.com (L.M.J.); Sj.spriewald@gmx.de (S.S.); stecher@mvp.lmu.de (B.S.); 3 Center for Infection Research (DZIF), Partner Site Munich, Pettenkoferstr. 9A, D-80336 Munich, Germany; 4 German Cancer Consortium (DKTK), Partner Site Munich, Marchioninistr. 15, D-81377 Munich, Germany; 5 Biobank under the Administration of the Human Tissue and Cell Research (HTCR), Marchioninistr. 15, D-81377 Munich, Germany

**Keywords:** gut microbiome, 16S rRNA, colorectal cancer, biobank, biorepository

## Abstract

**Simple Summary:**

The role of gut microbiota in colorectal cancer is subject to extensive research. The aim of this study was to assess the feasibility of DNA extraction and microbiome profiling of samples from different sample sites, tissue sites and storage duration of a colorectal cancer biobank. Mucosa samples, mucosal scrapings and feces as well as different tissue sites (tumor, normal mucosa) were analyzed. Microbiome analysis could be successfully performed in most of the samples (overall 93.3%) with sufficient numbers of high-quality reads. There were no differences between sample sites, while in some measures, significant differences were found between tumor and normal mucosa. Samples stored for up to eight years were used and storage conditions had no significant influence on the results. Microbiome analysis can be carried out successfully in fecal, normal mucosal and tumor samples stored long term in a colorectal cancer biobank, hence large retrospective microbiome association studies are feasible.

**Abstract:**

The role of gut microbiota in colorectal cancer is subject to extensive research. Before usage of biorepositories for microbiome studies, it is crucial to evaluate technical feasibility of microbiome profiling from various biospecimens. The aim of this study was to assess the feasibility of DNA-extraction and microbiome profiling of samples from different sample sites, tissue sites and storage duration of a colorectal cancer biobank. Mucosa samples, mucosal scrapings and feces as well as different tissue sites (tumor, normal mucosa) were analyzed. 16S rRNA gene-based microbiome profiling with taxonomic assignment was performed on the Illumina MiSeq (Illumina, San Diego, USA) platform from stored snap frozen samples. For statistical analysis, α- and β-diversity measures, PCoA, permutational multivariate analysis of variance and graphical representation were performed. Microbiome analysis could be successfully performed in most of the samples (overall 93.3%) with sufficient numbers of high-quality reads. There were no differences between sample sites, while in some measures significant differences were found between tumor and normal mucosa (α-diversity, Shannon/Simpson Indices *p* = 0.028/0.027, respectively). Samples stored for up to eight years were used and storage conditions had no significant influence on the results. Tumor and tissue samples of a biobank stored long term can be successfully used for microbiome analysis. As large sample sizes are needed for association studies to evaluate microbial impact on tumorigenesis or progression of colorectal cancer, an already established biorepository may be a useful alternative to prospective clinical studies.

## 1. Introduction

In recent years, the relevance of sequence-based analysis of the gut microbiota in patients with various diseases has risen sharply [1]. Association studies have linked alterations of the gut microbiota with a variety of human diseases [2,3,4,5,6,7,8]. The abundance of different microbiota also varies highly among healthy individuals depending on environmental conditions, genetics, the host’s immune system, diet as well as infections or use of antibiotics [9,10]. In colorectal cancer (CRC), there is an increasing evidence suggesting that the gut microbiota are associated with CRC development [11,12]. Gut microbiota show significant impact on processes involved in cellular DNA damage, DNA methylation, chromatin structure modulation and non-coding RNA expression [1]. Furthermore, they play distinct roles in pathways of cell proliferation such as WNT signaling [1,11]. *Fusobacterium nucleatum* was causally linked with tumorigenesis of sporadic CRC [13]. Furthermore, *Escherichia coli* strains harboring a genomic virulence island (*pks*) can cause DNA damage and chromosomal instability in the host [14]. In addition, next-generation sequencing technologies also revealed the relevance of other CRC-associated gut bacteria such as *Bacteroides fragilis*, *Escherichia coli*, *Parvimonas micra*, *Peptostreptococcus stomatis* and *Atobobium parvulum* [11]. According to the oral-gut hypothesis of CRC tumorigenesis, several of these bacteria, *Fusobacterium nucleatum*, *Parvimonas micra* and *Peptostreptococcus stomatis* in particular, originate from the oral cavity and are opportunistic colonizers of the colon reflecting intestinal dysbiosis [15,16]. Despite evident regional differences in microbial communities [17], Wirbel et al. could identify 29 microbial species consistently associated with CRC tumorigenesis throughout different countries [12]. Microbial signatures were even shown to improve sensitivity of CRC prediction when combined with fecal occult blood testing (FOBT) by >45% relative to FOBT alone [18]. In addition, microbial pathogens, such as *Enterobacteriaceae*, *Enterococcus* spp., *Staphylococcus* spp. and *Pseudomonas aeruginosa* have been shown to be associated with surgical complications, especially surgical site infections and anastomotic leakage in patients undergoing colorectal resections [10,19]. Hence, a better understanding of the gut microbiome might play an important role to improve diagnostics and therapy of CRC patients.

For researchers, the opportunity to analyze not only biospecimens within prospective or cross-sectional studies but also the chance to use large biorepositories would enable meaningful translational studies further investigating the role of the gut microbiota in CRC. For this purpose, biobanks can provide important resources, for example tumor and adjacent normal tissue samples, blood, patient-derived in vitro cell models as well as the corresponding clinical data. Identifying distinct phenotypes including the microbial profile could help to develop tailored diagnostic as well as therapeutic strategies depending on the individual risk for perioperative complications and cancer progression.

To enable the use of large colorectal cancer biorepositories for microbiome association studies, this work aimed to investigate the feasibility of microbiome analysis in snap frozen samples from a colorectal cancer biobank collected from different sample sites (mucosal, luminal), tissue sites (tumor, normal mucosa) and with varying storage durations.

## 2. Results

### 2.1. Microbiome Profiling of Luminal and Mucosal Sample Sites (Step 1)

The procedural characteristics from samples of step 1 experiments are shown in Table 1.

The samples were stored for an average time of 4.0 ± 1.3 months. Microbiome analysis could be successfully performed in five out of six fecal samples, five out of six mucosa samples and six out of six mucosal scraping samples (16 out of 18 samples overall, 89%) with a sufficiently high amplicon DNA concentration. Only in one fecal and one tissue sample, the amplicon DNA concentration was quite low despite a high DNA concentration after extraction (presumably human DNA). DNA yield after extraction and amplification as well as high-quality reads of samples are given in Table 2.

The mean DNA concentration of all samples after 16S rRNA gene amplification before sequencing was 5.38 ± 3.06 ng/µL (range: 0.04–10.18 ng/µL). After sequencing and quality control, the mean number of high-quality reads was 39,052 ± 27,173 counts/sample (range: 173–84,086 counts/sample). Because of a low number of high-quality reads in the above described fecal and tissue sample (173 and 249 reads/sample, respectively), these two samples were excluded from further analysis. In the remaining samples, 702,508 16S rRNA gene sequences could be obtained in total and sub-sampling (rarefaction) was performed to the size of the smallest amount of counts/sample (*n* = 4733) for each sample. There was a moderate inverse correlation between the number of obtained high quality reads per sample and concentration of genomic DNA after extraction (r = -0.476; *p* = 0.046) as well as a moderate correlation to the DNA concentration after amplification (r = 0.477; *p* = 0.045).

**Figure 1:**
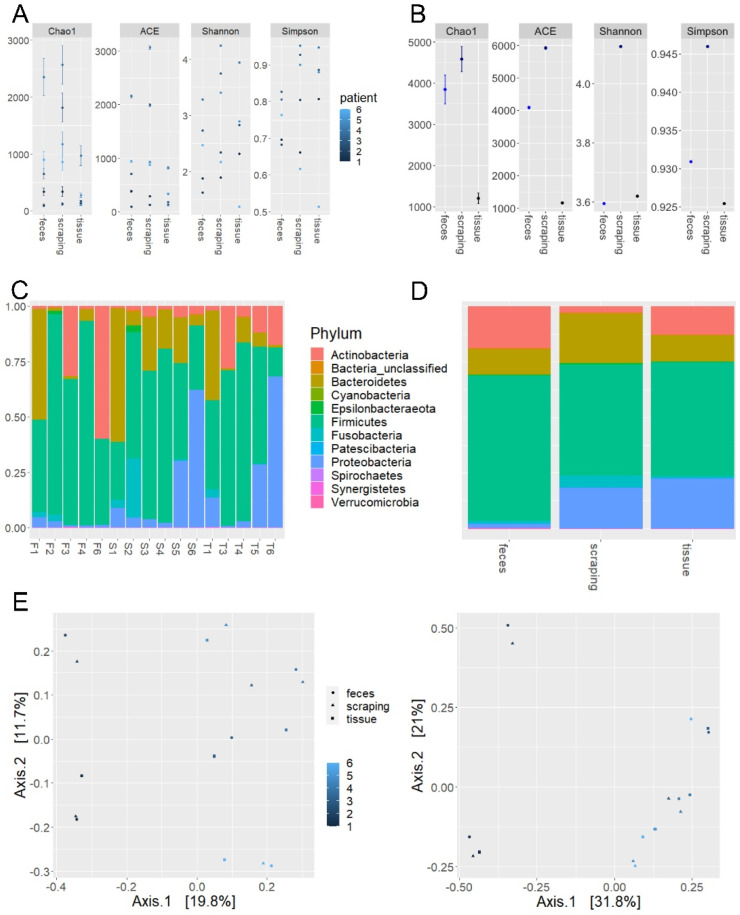
Alpha-diversity measures for all samples from (**A**) feces, scrapings as well as mucosa tissue and (**B**) between sample collection sites of trial step 1 experiments. Abundance on phylum level for (**C**) all individual samples from feces [F], scrapings [S], mucosa tissue [T] and (**D**) the same samples stratified according to sample site (number of reads normalized using median sequencing depth). Principal coordinate analysis based on weighted (**E**) and un-weighted (**F**) UniFrac distances for fecal samples, tissue samples and mucosal scrapings.

The α-diversity measures for mucosa samples, mucosal scrapings and feces of step 1 experiments are presented in Table 2 as well as Figure 1A,B. Wilcoxon rank sum test showed no significant difference between groups for α-diversity measures (Chao1 and ACE Richness, Shannon and Simpson Indexes; *p* > 0.05, pairwise comparison).

Predominant phyla were Firmicutes, Bacteroidetes, Proteobacteria, Actinobacteria and Fusobacteria (Table 3).

The relative abundance for the different samples based on phylum levels is shown in Figure 1C,D. The relative distribution of phyla showed variations to some extent, but due to small sample size no comparative analysis were performed. Weighted and unweighted UniFrac analyses showed no visual distinction between groups representing luminal and mucosal compartments (feces to scraping 0.43/0.74; feces to mucosa 0.36/0.69; scraping to mucosa 0.31/0.73) as shown by PCoA in Figure 1E,F. This could be confirmed by PERMANOVA (weighted UniFrac: R2 = 0.106; *p* = 0.925; unweighted UniFrac: R2 = 0.107; *p* = 0.681).

### 2.2. Microbiome Profiling from Tumor and Adjacent Normal Mucosa Samples as Well as Influence of Storage Duration (Step 2)

The procedural characteristics from samples of Step 2 experiments are shown in Table 1. The samples were stored for an average time of 58.5 ± 37.6 months. Microbiome analysis could be successfully performed with a sufficient amount of high-quality reads (836,791 16S rRNA gene sequences) in all six tumor and six corresponding mucosa tissue samples (12 out of 12, 100%) (Table 4).

Sub-sampling (rarefication) was performed to the smallest number of counts/sample (*n* = 15,147) for each sample. There was no relevant correlation between number of high-quality reads and duration of storage at −80 °C (r = 0.221; *p* = 0.490) or weight of the used samples (r = 0.123; *p* = 0.704).

**Figure 2:**
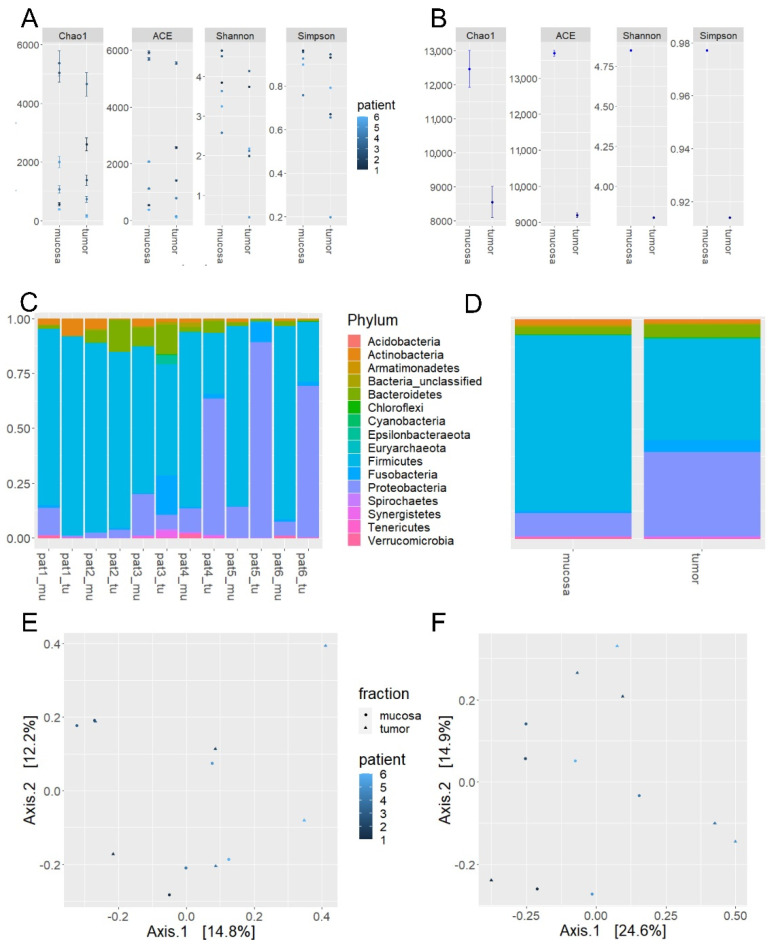
Alpha-diversity measures for all samples from (**A**) normal mucosa and tumor tissue as well as (**B**) between tumor and normal mucosa (mean) of step 2 experiments. Abundance on phylum level for (**C**) all individual patient samples [pat] and (**D**) the same samples grouped as tumor or mucosa tissue (number of reads normalized using median sequencing depth; tu = tumor sample; mu = mucosa sample). Principal coordinate analysis based on weighted (**E**) and un-weighted (**F**) UniFrac distances for mucosa and tumor samples.

Comparing the α-diversity measures between mucosal and tumor site samples, no significant differences between groups for Chao1 (*p* = 0.249) or ACE (*p* = 0.249) richness-based measures was observed; for Shannon and Simpson indices, respectively, there were significant differences between groups (*p* = 0.028 and *p* = 0.027, respectively, Wilcoxon rank sum test; Table 4, Figure 2A,B).

Predominant phyla are shown in Table 4 and Figure 2C,D. Actinobacteria were significantly more abundant in normal mucosa samples compared to tumor tissue (*p* = 0.028, Wilcoxon rank sum test). Beyond that, a variation in the relative distribution of phyla between mucosa and tumor samples was observed to some extent without significance with e.g., a higher proportion of Fusobacteria in tumor tissue samples as compared to mucosa samples.

Weighted and unweighted UniFrac analyses showed no visual distinction between tumor and normal mucosa samples representing the mucosal compartment (mucosa to tumor 0.795/0.528) as shown using PCoA in Figure 2E,F. This was confirmed by PERMANOVA (weighted UniFrac: R2 = 0.0831; *p* = 0.758; unweighted UniFrac: R2 = 0.10783; *p* = 0.263).

**Figure 3:**
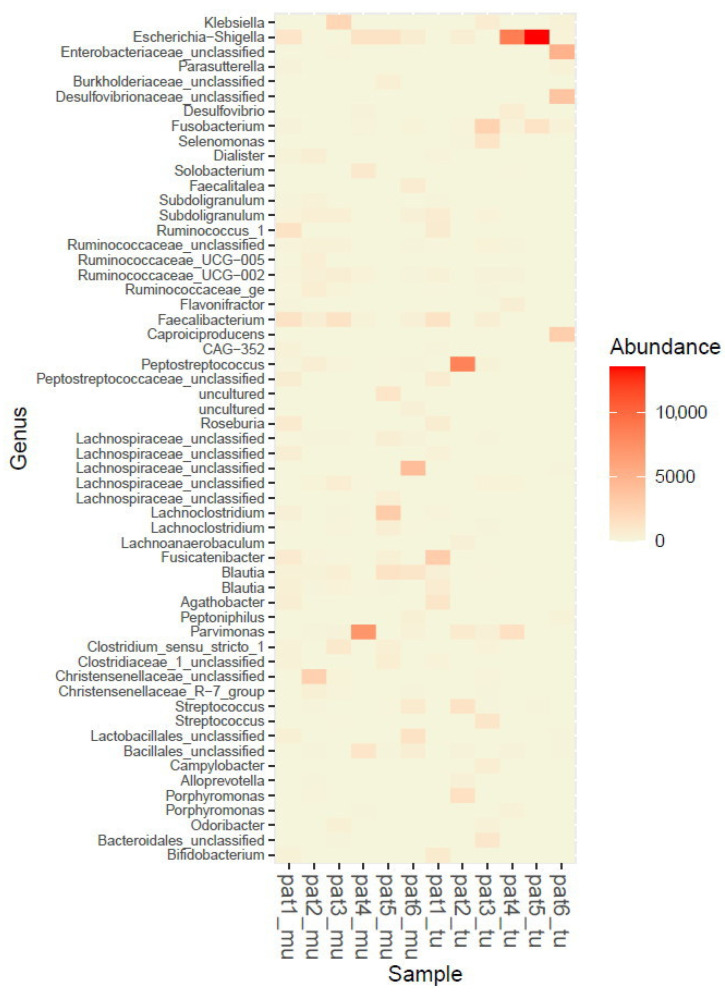
Heatmap of abundance at the genus level for all samples (pat = patient, mu = mucosa, tu = tumor samples).

**Figure 4:**
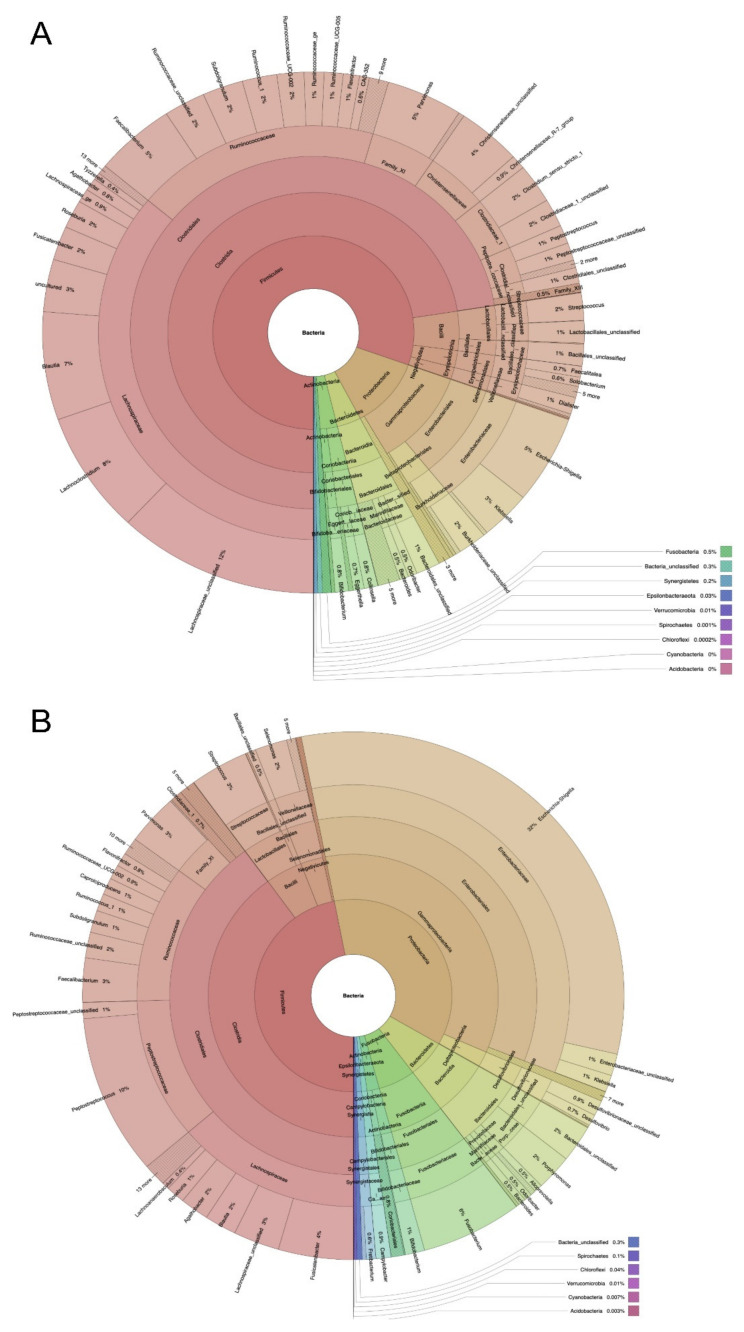
Krona diagrams for illustration of abundance of different OTUs at the genus level (**A** mucosa, **B** tumor).

The abundance of mucosa and tumor samples is also illustrated as heatmap (Figure 3) and krona diagrams (Figure 4A,B).

## 3. Discussion

In CRC, the causative role of microbiota in tumorigenesis and progression is widely accepted [11,12,20,21]. However, despite the significant impact of microbiota on CRC development and progression as well as outcomes after colorectal surgery, the role of the microbiome and its interaction with the host is still not fully understood and requires further research. Several studies revealed associations between the colonization of specific species and tumor-specific characteristics such as lymph node metastasis and gene mutations (summarized by Chen et al. [20]). Comparisons of taxonomic groups based on phyla of healthy people and CRC patients revealed differences in the abundance of *Proteobacteria*, *Bacteroidetes*, *Actinobacteria*, *Firmicutes* and (to the greatest extent) *Fusobacteria* [20]. Furthermore, some *Escherichia coli* strains harboring the *pks*-island (Colibactin gene), enterotoxigenic *Bacteroides fragilis* and *Fusobacterium nucleatum* showed the ability to induce a proinflammatory microenvironment, cell proliferation and even direct DNA damage [11,12,22,23,24,25].

As gut microbiota significantly contribute to immune function and the restoration of body integrity in healing processes, its impact on wound and anastomotic healing after surgery is under evaluation [26,27]. There are complex interactions between gut microbiota and essential cytoprotective mechanisms, bacterial metabolites as well as the immune system. The restoration of a healthy intestinal microbiome following colorectal surgery is depending on multiple perioperative variables such as extent of surgical trauma, perioperative use of antibiotics, bowel preparation and possibility of oral nutrition [19,26]. The use of minimally invasive surgical technique, limited use of antibiotics and fast return to oral intake of intake of unprocessed food may have beneficial effects on the intestinal microbiome, whereas refaunation by pathogenic bacteria like *Serratia* spp., *Enterococcus* spp. and *Pseudomonas* spp. following colorectal surgery may significantly increase the risk of anastomotic leakage and surgical site infections as well as sepsis [10,26].

Given the relevance of the microbial profile in CRC and its impact on short-term outcome following colorectal surgery, the mechanisms of interaction between the microbiota and the gut epithelium as well as mucosa-associated immune tissue highlight the importance of microbiome analysis of the mucosal microbial compartment. The results of the present work show that microbiome profiling can be successfully conducted from different compartments and tissue sites, even after long-term storage of snap-frozen samples. In order to choose the right sample type for microbiome studies, one must take into account the differences in the composition of different microbial compartments and tissue sites. We have shown that these sample types could be carefully chosen from samples stored in a comprehensive biobank in a retrospective manner depending on the specific scientific aim.

As fecal samples (luminal compartment) are easy to collect in a non-invasive manner, they are currently widely used for microbiome profiling in association studies, especially for colorectal cancer [11,12]. Taking into account differences in microbial patterns between different anatomical locations of the colon not only in CRC, but even healthy individuals, the fecal microbiome appears to be more appropriate for an use as biomarkers as it is representing the overall microbial composition of the colonic microenvironment [16,28]. However, the fecal microbiota are more inconsistent due to a higher impact of environmental factors like diet or life-style on the luminal micromilieu [25,29]. The mucosal microbiota are more stable, but can only be analyzed from biopsies or surgical specimen; they are located at the direct interface with the gut epithelium and consequently, are more closely involved in immune processes [25,29,30,31]. Therefore, the mucosal microbiota play an essential role in chronic inflammation and tumorigenesis in CRC due to their direct interaction with epithelium and immune cells at the gut-mucosa barrier [11,12,22,25]. Codling et al. revealed that the intra-individual similarity between luminal and mucosal compartments in a larger cohort was in a range of 84% [29]. In the present work, we show some variations in phylum level abundance between the compartments without relevant significance in diversity measures or weighted/unweighted UniFrac analysis.

Comparing the microbiome profiles of tumor to off-tumor-site tissues, we observed a significant reduction in α-diversity indices for tumor tissue, which is consistent to recent publications and reflects the intestinal dysbiosis associated with CRC [32]. Furthermore, we could detect some variation in phylum level abundance. Actinobacteria were significantly more abundant in samples of normal mucosa, whereas a non-significant higher abundance of Fusobacteria was observed in tumor tissue samples. These results are in accordance to previously published data [12,17,22,24,25] and supporting the role of *Fusobacterium nucleatum* as a “passenger” in the tumorigenesis of CRC according to the widely accepted “driver-passenger mode” proposed by Tjalsma et al. [33].

In general, our microbiome profiling results are comparable to other available data using samples from healthy controls and patients with CRC. The predominant phyla were *Firmicutes*, *Proteobacteria* and *Bacteroidetes*, whereas *Actinobacteria* and *Fusobacteria* were less abundant [20,24,25]. Recently published data examined the microbial niches along the gastrointestinal tract in German healthy individuals and could demonstrate differences especially between upper and lower gastrointestinal tract and even between the different anatomical regions of the colon and also compared to fecal samples [28]. The predominant phyla in the colon and feces were *Firmicutes*, *Bacteroidetes*, *Proteobacteria* and much less abundant *Fusobacteria* [28]. An analysis of mucosa-associated microbiome profiles in colorectal cancer samples showed that *Firmicutes*, *Bacteroidetes* and *Fusobacteria* were the most abundant phyla and *Proteobacteria* were less common in these samples [24]. The α-diversity measures like Shannon or Simpson indices show some variations in the available data due to methodical and maybe even regional differences [17], but are consistently showing reduced α-diversity levels in CRC in accordance to evident dysbiosis as a main finding in microbial patterns of CRC samples [22,24,25]. Therefore, compared to other available data and taking into account regional and inter-individual differences in abundance of phyla and other taxonomic levels, our technique of sampling, DNA extraction, DNA amplification, sequencing and bioinformatic analysis revealed valid microbiome profiles of the different compartments and tissue samples [16,24,25,28].

In addition, the results of the present work show that valid microbiome profiling even from long-term stored snap frozen samples can be successfully conducted from different compartments and tissue sites. The storage duration of our samples did not significantly affect the number of high-quality DNA reads. The samples were stored at −80 °C for a mean of five years, some of them for longer than eight years. Nonetheless, it was possible to perform high-quality microbiome profiling on these samples. All samples were snap frozen in liquid nitrogen with feasibly short ischemia times according to established protocols and then stored at our biobank facilities in concordance with previously published recommendations [34,35]. An established high-volume colorectal tissue repository based on an ethical and legal framework [36,37] therefore represents a suitable base for microbiome research.

The limitations of our work include the number of samples and some variances in biobank and analysis processes. Samples from surgical specimens were immediately collected after removal of the specimen from the surgical field during surgery, but some factors such as warm ischemia or perioperative antibiotic prophylaxis might differ between samples. In this analysis we did not include samples of rectal cancer tissues as in rectal cancer surgery more confounding factors would have had an impact on microbiome analysis due to especially mechanical bowel preparation and neoadjuvant radio-chemotherapy. The use of samples originating mostly of the proximal colon without perioperative use of mechanical bowel preparation allowed us for maintaining an as homogeneous as possible cohort and reduce gross interindividual differences as well as evident differences between anatomical sample sites [16,28].

A comparison to microbiome profiles from stool and tissue samples of other available studies indicates that our results might be of some external validity [20,24,25,29]. Despite high inter-individual differences in the gut microbiome, the results of many microbiome studies are limited by small sample sizes and this limitation is also inherent in our study. Nonetheless, our work provides the basis for future work by proving that a retrospective approach assessing a high number of stored samples is feasible and valid microbiome profiles can be generated using long term stored samples.

## 4. Materials and Methods

### 4.1. Ethical Framework

This study was approved by the Ethics Committee of the Faculty of Medicine, Ludwig-Maximilians-University (LMU), Munich, Germany. Double-coded tissue and stool samples as well as corresponding procedural and clinical data used in this study were provided by the Biobank of the Department of General, Visceral and Transplant Surgery, LMU, under the administration of the Human Tissue and Cell Research (HTCR^®^) Foundation. The framework of the HTCR Foundation includes obtaining written informed consent from all donors and has been approved by the Ethics Committee of the Faculty of Medicine, LMU, Munich, Germany, (approval number 025-12) as well as the Bavarian State Medical Association (approval number 11142), Germany [36].

### 4.2. Study Design and Sample Collection

From patients undergoing right or left hemicolectomy for colorectal cancer, gut microbiome 16S rRNA gene-based microbiome analysis was performed in two independent trial steps. After initial macroscopic examination of the specimen by an expert pathologist, tissue for sample collection was transferred to the biobank’s laboratory. Samples from six donors from different sample sites such as feces (luminal compartment), mucosa samples and mucosal scrapings (mucosal compartment) were prospectively collected in the first trial step (step 1) between March and July 2018. Samples were stored snap-frozen using DNA free Eppendorf tubes (2.0 mL DNA LoBind Tubes, PCR clean, Eppendorf, Hamburg, Germany) at −80 °C. In a second trial step (step 2), microbiome profiling of samples of right or transverse colon from different tissue sites (tumor or normal adjacent mucosa from the same surgical specimen) was performed to assess the mucosal microbiome of tumor and normal adjacent mucosa as well as to investigate the influence of varying storage durations. Therefore, snap-frozen tissue samples of six further donors were used, which were stored at −80 °C at the biobank between 2010 and 2018 [34,36,37].

### 4.3. DNA Extraction, Amplification and Sequencing

Each sample was suspended in 500 μL of extraction buffer (200 mM Tris pH 8.0, 200 mM NaCl, 20 mM EDTA), 210 μL of 20% SDS, 500 μL of a mix of phenol:chloroform:isoamyl alcohol (24:24:1) and 500 μL of zirconia/silica beads (0.1 mm diameter). Lysis of bacterial cells was obtained by mechanical disruption with a bead beater (50 s^−1^) for 4 min, followed by DNA extraction in phenol:chloroform:isoamyl alcohol and precipitation with ethanol. The isolated DNA was resuspended in 10 mM Tris buffer and purified with NucleoSpin gDNA clean-up columns (Macherey-Nagel, Düren, Germany) in a final elution volume of 50 µL. The extracted DNA was amplified by PCR using multiplexed 8 forward x 12 reverse-primer, specific for the variable V3-4 regions of the 16S rDNA gene (5′-CCTACGGGNBGCASCAG-3′ and 5′-GACTACNVGGGTAT CTAATCC-3′). The amplicons were purified using the Agencourt AMPure XP PCR Purification system (Cat: A63880, Beckman Coulter, Krefeld, Germany). Library quality control and DNA sequencing on the Illumina MiSeq v.3 platform (300-bp paired-end runs) were performed at Eurofins Genomics (Ebersberg, Germany).

### 4.4. Bioinformatics and Statistical Analysis

The sequencing output was processed and joined reads were then quality filtered during demultiplexing using QIIME v1.8 to retain only high-quality reads [38]. Only reads that possessed the full barcode free of errors, were longer than 200 bp after removal of the variability region as well as quality trimming and had a minimum of 30 bp overlap without mismatches during merging of paired-end reads were kept for further analysis.

Chimeric sequences were removed with USEARCH v6.1 (step 1) [39] and vsearch (step 2) [38]. respectively. Open-reference OTU clustering and taxonomic assignment using paired-end sequencing reads was done against the Silva database release 132 at the 97% similarity level using mothur v1.43.0 [40]. Further analysis was performed using *R* studio (version 1.8 and phyloseq package [41]) as well as SPSS (IBM, Armonk, NY, USA; Version 25). For analysis of diversity measures, ordination and non-parametric analyses, data were sub-sampled to the sample with the lowest number of reads. For diversity analysis, α-diversity measures including Chao1 richness, abundance-based coverage estimators (ACE) and diversity indices Shannon and Simpson Index were calculated. For estimation of differences between groups, weighted and unweighted UniFrac were calculated as distance metrics [42,43]. Based on UniFrac distances, principal coordinate analysis (PCoA) was performed for graphic illustration. Permutational multivariate analysis of variance (PERMANOVA) was preformed using *R* vegan [44] package (ADONIS function) for analysis of dissimilarities between groups based on UniFrac distance metrics [42,43]. Heat maps and bar as well as box plots were generated using the *R* ggplot2 package [45]. For comparison of diversity measures and abundance of most abundant phyla between groups, Wilcoxon rank sum test and Friedman two-way analysis of variance were used. Pearson correlation was used to analyze the relationship between genomic DNA concentration after extraction, amplicon concentration, storage conditions and number of high-quality reads per sample. Krona diagrams of composition of local microbial environment were generated using Krona tools [46]. *p* values < 0.05 were considered statistically significant.

## 5. Conclusions

The results of the present work show that microbiome analysis, even from long-term stored snap frozen samples of a colorectal cancer biorepository, can be successfully conducted from different compartments and tissue sites. For several scientific study aims, this may be a feasible approach avoiding the time-consuming prospective collection of samples to perform association studies based not only on microbiome research but also to assess metabolomics profiles in CRC [23].

## Figures and Tables

**Table 1:** Procedural characteristics of samples collected from different sample (Step 1 experiments) and tissue sites as well as storage duration (Step 2 experiments).

	Sample Collection Site	Weight (g) of Samples (Mean)	IT ^1^ (*min*) OR ^2^ until Sampling (Mean ± SD)	IT ^1^ (*min*) Sampling until Freezing (Mean ± SD)	Duration (Months) of Storage at −80 °C (Mean ± SD)
Step 1 n = 6	Feces	0.19	36.3 ± 23.8	114.3 ± 95.6	4.0 ± 1.3
	Mucosa	0.23			
	Scraping	0.36			
Step 2 n = 6	Mucosa	0.20	39.2 ± 20.3	37.8 ± 10.0	59.0 ± 37.6
	Tumor	0.26			

^1^ ischemia time; ^2^ operation room.

**Table 2:** DNA, amplicon yield, amount of high-quality reads and α-diversity measures in samples collected from different compartments (Step 1).

		Basic Data			α-Diversity Measures			
		gDNA (ng/µL) (PicoGreen)	DNA Concentration (ng/µL) after Amplification	Reads/Sample	Chao1	ACE	Shannon	Simpson
Feces	1	103.88	9.95	38,830	3848	4094	3.59	0.93
	2	186.72	7.98	61,629				
	3	143.18	4.23	4733				
	4	163.34	3.26	54,584				
	5	165.46	0.04	249 *				
	6	116.20	6.32	74,427				
	Mean ± SD	146.5 ± 31.6	5.30 ± 3.50	39,075 ± 30,613				
Mucosa	1	182.15	3.17	46,077	1207	1163	3.62	0.93
	2	172.71	0.27	173 *				
	3	174.29	3.60	6058				
	4	167.36	5.82	8294				
	5	179.41	7.84	13,265				
	6	171.14	5.32	38,225				
	Mean ± SD	174.51 ± 5.45	4.34 ± 2.60	59,397 ± 14,974				
Scraping	1	18.95	7.25	62,021	6165	6064	4.08	0.95
	2	42.91	1.68	45,152				
	3	8.14	6.65	50,863				
	4	118.70	8.98	84,086				
	5	6.86	10.18	46,675				
	6	6.04	4.33	67,589				
	Mean ± SD	33.60 ± 43.97	6.51 ± 3.11	18,682 ± 18,823				

* Failed samples.

**Table 3:** Relative abundance for most abundant species on phylum level (%) in step 1 experiments.

Species	Feces	Scrapings	Mucosa
*Firmicutes*	65.8	50.4	51.4
*Bacteroidetes*	11.6	22.1	12.1
*Proteobacteria*	2.1	18.5	22.7
*Actinobacteria*	18.9	2.9	12.9
*Fusobacteria*	1.1	5.2	0.8

**Table 4:** Amount of high-quality reads and α-diversity measures as well as relative abundance for most abundant species on phylum level (%) of tumor and normal mucosa tissue samples in Step 2 experiments.

		Mucosa	Tumor
Reads/sample ± SD		70,621 ± 30,690	68,843 ± 29,020
α-diversity measures	Chao1	12,465	8553
	ACE	13,690	9191
	Shannon	4.85	3.80
	Simpson	0.98	0.91
Relative abundance	*Firmicutes*	80.5	46.3
	*Proteobacteria*	10.9	38.6
	*Bacteroidetes*	3.6	5.9
	*Actinobacteria*	2.6	1.8
	*Fusobacteria*	0.6	5.2
